# Supplementary material for: The role of albumin-corrected anion gap as a predictor of all-cause mortality in patients with Sepsis-AKI: A propensity score-matched cohort study
Source: PLoS One. 2025 Jul 18;20(7):e0327914. doi: 10.1371/journal.pone.0327914 (PMC12274010; doi:10.1371/journal.pone.0327914)
Supplement: Attachment — Submitted filename: Response to Reviewers.docx [file pone.0327914.s002.docx]

Response to reviewers

Dear Editor and reviewers,

Thank you for reviewing our manuscript and for the constructive comments, which greatly helped us to improve the manuscript (PONE-D-24-27578). We appreciate the promoting comments to our study, and we have accepted and revised as recommended in this revised manuscript. We highlighted all the revisions in yellow/red colour.

We would like to express our great appreciation to you and reviewers for comments on our paper. Looking forward to hearing from you.

Thank you and best regards.

The main corrections in the paper and the responds to the comments are as following:

**Additional Editor Comments:** In this manuscript where authors analyzed the role of albumin corrected anion gap in sepsis related AKI, authors should specifically discuss what albumin correction will add in following patients who already have high anion gap. The utility of albumin correction should be given.Also, I recommend that you check your data. E.g. Decimals of Na and BMI were not given and albumin levels are all same for groups in table 3. Reliability of data is highly crucial as our publication criteria.

**Response:** 1、Thanks to the editor’comment. A high AG is often associated with severe clinical prognosis, especially in patients with sepsis. Patients with sepsis often have hyperlactatemia and metabolic acidosis, resulting in increased AG, but patients with sepsis are often accompanied by hypoalbuminemia. Since albumin is negatively charged that will lead to a decrease in AG,hypoalbuminemia may lead to false negative AG results, affecting the accurate judgment of the results. Therefore, the ACAG was used to more accurately reflect the presence of unmeasured anions by adjusting AG based on albumin levels. We have supplemented this in the Discussion section.

2、Na in MIMIC IV is an integer. We conduct statistical analysis again and correct the contents of the table. In actual operation, even after PSM, it is possible that the matched groups still have differences in some indicators, or are exactly the same. This depends on the characteristics of the data itself, the matching algorithm chosen (such as nearest neighbor matching, caliper matching, etc.), the stringency of the matching (such as the size of the caliper value), and the size of the sample size. If the matched two groups are exactly the same on a certain metric, this may mean that the two groups themselves are similar on that metric, or that the matching algorithm was very efficient at finding similar control groups.

**Reviewer #1:**

**Comment 1:**There is no data provided on causes of acute kidney injury (AKI) such as drugs, toxins, and other factors. These data should be included, and you should statistically analyze these contributing factors.

**Response:** I would like to extend my gratitude for your valuable feedback on our manuscript. Your suggestion regarding the inclusion of data on the causes of AKI, such as drugs, toxins, and other contributing factors, is indeed insightful.We recognize that incorporating data on the specific etiologies leading to AKI would enhance the depth and precision of our analysis. However, our study relied on the MIMIC-IV database, a publicly accessible and widely utilized clinical database that, while extensive, does not encompass all possible clinical details, including specific causes of AKI like particular medications or toxin exposures. Our choice of the MIMIC-IV database was driven by the breadth and diversity of the data it offers, allowing us to investigate general trends and outcomes in AKI, which is crucial for understanding the disease's impact and improving patient prognosis. Although our study has its limitations, we believe that the analysis of the relationship between the ACAG and AKI prognosis provides clinicians with a useful prognostic tool.

We concur that future research should consider including these data. We recommend that subsequent studies might utilize other databases that contain more detailed information or conduct prospective studies designed to collect such data. We are also open to exploring these factors in our future research endeavors.Once again, we appreciate your suggestions and look forward to further discussions, hoping to address your concerns adequately.

**Comment 2:** How did you classify acute kidney injury? You need to explain the criteria related to AKI according to the KDIGO guidelines.

**Response:** Thanks for the reviewer’s comment. The definition criteria of AKI have been supplemented according to KDIGO (references 19). We have mentioned this in the part of Methods marked in yellow.

**Comment** **3:** Please provide details on how patients were diagnosed with AKI stages I, II, and III. How were the patients classified into these AKI categories?

**Response:** When extracting information on patients diagnosed with AKI from the MIMIC IV database, the stage of AKI was included. Perform a literature search using “MIMIC IV” as the keyword and we can find that many studies have extracted information on AKI and staging. We have made corresponding additions to make the article more complete.

**Comment** **4:** The definition of sepsis needs to be more clearly defined. The sentence "Sepsis is a syndrome characterized by life-threatening organ dysfunction and is a frequently encountered serious complication of critical illnesses such as trauma, infection, and shock" should be more comprehensive.

**Response:** Thanks to the reviewers’comments, we have refined the definition of sepsis in the **Introduction** section.

**Comment 5:** For the inclusion criteria, you wrote "patients with Sepsis-induced AKI (SOFA score ≥ 2 and also suspected of infection) during ICU admission." Is this the correct definition of sepsis-induced AKI? I am not familiar with this terminology in the literature. Please review this point.

**Response:** Thank you for your insightful comments and for drawing our attention to the need for a more detailed definition of Sepsis-Associated AKI in our manuscript. In response to your comment, we have now included a more detailed explanation of Sepsis-AKI in the Methods section of our manuscript. We have referenced the Kidney Disease: Improving Global Outcomes (KDIGO) clinical practice guidelines.The guideline defined AKI as an abrupt decrease in kidney function occurring over 7 days or fewer: increase in SCr by ≥ 50% within 7 d, or increase in SCr by ≥ 0.3 mg/dL ( ≥26.5 mmol/l) within 48 h, or oliguria.

**Reviewer #2:**

**Comment 1:** Operational definition of sepsis AKI need to be mentioned details in methodology.

**Response:** Dear Reviewer,Thank you for your valuable feedback and for pointing out the need to clarify the definition of Sepsis-AKI in our manuscript.In line with the comments made by reviewer#1, we have added the definition of Sepsis-AKI in the methods section.

**Comment 2:** Do you think table before and after PSM analysis required.

**Response:** We appreciate your question regarding the necessity of including tables that display the analysis before and after Propensity Score Matching (PSM). We believe that presenting both tables serves multiple important purposes in our study.**1.Transparency:** Showing the data before PSM allows readers to see the original distribution of variables and any potential confounding factors that might exist between groups.**2.Assessment of PSM Effectiveness:** The comparison of the tables before and after PSM demonstrates the effectiveness of the matching process in balancing covariates between groups. This helps in validating the robustness of our results.**3.Context and Interpretation:** The before-and-after presentation provides context for the changes that occur as a result of the PSM, allowing readers to interpret the impact of the matching on the study outcomes.**4.Completeness:** Including both tables ensures that our report is comprehensive and thorough, which is particularly important for studies utilizing complex statistical methods like PSM. We have carefully considered an alternative approach where we might only present the PSM results, but we concluded that the full presentation better serves the readers by providing a complete picture of the study's methodology and findings.

**Comment 3:** To add more literature on discussion about similar findings in other cohort such as shock. pancreatitis, trauma, or any other causes of well-known metabolic acidosis.

**Response:** We have added References 23 and 24 in the **Discussion**

section.

Dear Editor and reviewers,We hope that the revision is acceptable, and your favorable consideration of our manuscript is greatly appreciated. Best regards.

Jian Liao

2024-10-07

Discussion marked in red.e mentioned this in the last paragraph of Discussion marked in
