# Supplementary material for: The role of albumin-corrected anion gap as a predictor of all-cause mortality in patients with Sepsis-AKI: A propensity score-matched cohort study
Source: PLoS One. 2025 Jul 18;20(7):e0327914. doi: 10.1371/journal.pone.0327914 (PMC12274010; doi:10.1371/journal.pone.0327914)
Supplement: Attachment — Submitted filename: Response to Reviewers (2).docx [file pone.0327914.s003.docx]

Response to reviewers

Dear Editor and Reviewers,

We appreciate your comments and suggestions on this manuscript, which have enhanced its scientific rigor and readability.We are now proceeding with the second round of revisions.

**Reviewer #1:**

**Comment 2:** Recheck the tables: Just make sure the table format in line with journal format.

**Response:** We have readjusted the format of the table.

**Comment 3:** Recheck the citations and references: To make sure reference also in line with the journal format.

**Response:** We have readjusted the format of the references.

**Reviewer #2:**

**Comment 1:** I’ve noticed that some of the sections, particularly in the methodology and result sections, exhibit typographical errors, incorrect punctuation and lack of fluency which may affect clarity. I recommended a careful proofreading to ensure precision in the manuscript information.

**Response:** We have revised and polished the language in the Methods and Results sections to enhance the accuracy of the manuscript.

**Comment 2:** I suggest removing ‘the following reports provide further insights’ from the last statement in the final paragraph of the introduction section, as the arguments are already adequate.

**Response:** We have removed this sentence.

**Comment 3:** Could the author clarify the Sepsis-3 definition in the paragraph? Is it referring to the definition of Sepsis-AKI or sepsis in general?

**Response:** In the introduction section, we defined Sepsis-3, and in the methodology section, we defined Sepsis-AKI, citing relevant guidelines or literature.

**Comment 4:** Please rewrite the definition of KDIGO and the ethical part in a fluent manner.

**Response:** We have made readjustments in “study population” section and highlighted them in yellow.

**Comment** **5:** What does ‘exclusively acquired laboratory variables’ mean? Please rephrase, in order to add to a better understanding.

**Response:** We have made revisions again.

**Comment 6:** In the statistical part, may the author explain the reason of choosing p-value < 0.05 instead of p < 0.2 for multivariable Cox hazard regression? By choosing a p-value < 0.2, the author may ensure there is no important variable missing. PSM may eliminate cofounding by pairing similar characteristics but does not replace potential confounders that may be prematurely excluded.

**Response:** We sincerely appreciate the reviewers' valuable comments on our statistical section. We choosed p-value < 0.05 instead of p < 0.2 for multivariable Cox hazard regression due to the following reasons:

**Avoid Overfitting:** Retaining variables with p<0.2 in multivariable analysis might introduce noise and overfitting, compromising clinical interpretability and generalizability.

**Integration of Clinical Relevance:** In the final model, only variables with p < 0.05 were retained, and adjustments were made based on clinical expertise (such as known risk factors) to ensure that the selected variables were both statistically significant and biologically plausible.

Meanwhile, we have organized the data in Table 2 and Table 4 to avoid excessive data affecting readability.

**Comment 7**: May I know how the author checks for the balance of the data between matched cohorts?

**Response:** Standardized mean difference (SMD) values can also be used to assess the success of matching. Typically, an SMD of less than 0.1 for each variable is considered indicative of good balance in the cohort. We used P>0.05 to indicate that there was no statistical significance in other variables between the two groups, while the outcome measures were statistically significant, making it easier to understand and more readable.

We have provided additional explanations in the Results section, which have been highlighted in yellow.

**Comment 8**: For the result section, I would like to suggest a clear display of data by grouping the explanation on data pertaining to Table 1 and Table 3. Does Table 3 referring to the PSM data? Currently the titles of Table 1 and Table 3 are the same with similar variables. I also did not appreciate the explanation of Table 3 information, or any explanation comparing before and after cohort matching of variables.

**Response:** We appreciate the editor's comments on the inappropriate and unscientific presentation of the tables. We have merged Table 1 and 3 and newly included the SMD values, which allows for a more intuitive visualization of the differences in data before and after matching.The results section has been rephrased.

**Comment 9**:For the predictors, I would like to suggest that extracting the independent variables before and after PSM in a table, and kindly highlight any univariate variables that change in terms of significance after PSM. It is important to have data transparency, however data organisation is also vital to the reader. One way to address these is to submit the complete information of Table 2 and 4 as supplementary data.

**Response:** Thank you for the reviewers' suggestions. We have resubmitted the matched data as supplementary data.

**Comment 10**: I would like to suggest the author add a depth of discussion with literature evidence in the final part of the discussion. Now we know that ACAG>20 predicts all-cause mortality for sepsis-AKI patients. How do you relate it with clinical significance? As an example, will it helps in patient triaging? Will we now adopt a different approach in this population?

**Response:** Thank you for the reviewer's comments. We have supplemented the discussion section accordingly and highlighted the additions in yellow.
